# Underwater Acoustic Wavefront Visualization by Scanning Laser Doppler Vibrometer for the Characterization of Focused Ultrasonic Transducers

**DOI:** 10.3390/s150819925

**Published:** 2015-08-13

**Authors:** Roberto Longo, Steve Vanlanduit, Galid Arroud, Patrick Guillaume

**Affiliations:** 1 Vrije Universiteit Brussel, 1050 Brussels, Belgium; E-Mails: Steve.Vanlanduit@vub.ac.be (S.V.); Galid.Arroud@vub.ac.be (G.A.); Patrick.Guillaume@vub.ac.be (P.G.); 2 ESEO Group–GSII, 49107 Angers, France; 3 LUNAM Université, LAUM–CNRS UMR 6613, 72085 Le Mans, France

**Keywords:** underwater ultrasonic, scanning laser Doppler vibrometer, focused transducers

## Abstract

The analysis of acoustic wave fields is important for a large number of engineering designs, communication and health-related reasons. The visualization of wavefronts gives valuable information about the type of transducers and excitation signals more suitable for the test itself. This article is dedicated to the development of a fast procedure for acoustic fields visualization in underwater conditions, by means of laser Doppler vibrometer measurements. The ultrasonic probe is a focused transducer excited by a chirp signal. The scope of this work is to evaluate experimentally the properties of the sound beam in order to get reliable information about the transducer itself to be used in many kinds of engineering tests and transducer design.

## 1. Introduction

Visualization of acoustic wavefronts started to be the object of intensive research in the 1960s. The understanding of acoustic wavefronts and their interaction with objects is important for optimizing both the performance of acoustic sources and detectors and for the generation of structures, surfaces and materials with particular acoustic absorption and scattering characteristics. Moreover, the visualization of acoustic wavefronts represents a reliable test for transducer design or periodic control, *i.e.*, to check if the properties of the generated sound beam (such as sound field, focal zone, beam diameter; see Appendix A) still persist over a long-term period.

The gold standard technique for acoustic field characterization at MHz frequencies involves the use of hydrophones [1]. Hydrophones are special microphones able to measure acoustic fields with a spatial resolution less than 0.1 mm, but the possible movement of the probe along the scanning region could affect the acoustic field propagation. Moreover, experimental setup issues can arise in the case where there is limited room to place the microphone.

The most promising approach to develop new transducers capable of non-invasive visualizing acoustic wavefronts has been to consider optical metrology techniques. Specific examples of these methods can be identified as schlieren [2], Michelson interferometry [3], electronic speckle pattern interferometry (ESPI) [4] and laser Doppler anemometry (LDA) [5]. Michelson interferometry and LDA for acoustic analysis are both limited by the fact that they are mainly used as single-point techniques. Nevertheless, a possible employment of scanning Michelson interferometers has been introduced by Knuuttila *et al*. and can be found in [6]. Conversely, schlieren and ESPI are inherently whole-field in their analytical approach; however, schlieren has usually been used as a qualitative technique, and ESPI has demonstrated a poor signal-to-noise ratio (SNR) [7]. An alternative technique that has more recently been demonstrated is that of laser Doppler vibrometry (LDV). The applicability of LDV to the field of non-destructive testing (NDT) and experimental modal analysis (EMA) has been discussed in [8,9,10,11]. Many research activities count on LDV for wavefront visualization in air [12,13,14,15], solids [16] and water-based experimentation [17,18,19,20]. LDV measurements in water pose more problems compared to the ones in air. This is mainly due to the poor SNR of the acquired signals, which could impair the quality of the derived two-dimensional (2-D) images representing the ultrasonic wavefronts. Another problem for this kind of measurement is the presence of undesired multiple reflections within the water tank, due to the low attenuation coefficient of water.

In this article, it is our intention to improve and extend the use of underwater LDV measurements. The main objective is to quickly obtain high-quality images of the acoustic wavefronts, allowing us to experimentally determine the sound beam properties of a focused transducer. To reach this goal, a high-speed experimental setup with dedicated signal processing techniques to filter undesired multiple reflections has been developed. As LDV visualization of acoustic wavefronts represents an important topic in this paper, a brief theoretical abstract of this subject is proposed in the next paragraph. The method (including the experimental setup and the post-processing technique), as well as the results and conclusions will follow afterwards.

### Visualization of an Acoustic Wavefront by Means of LDV Measurements

**Figure 1:**
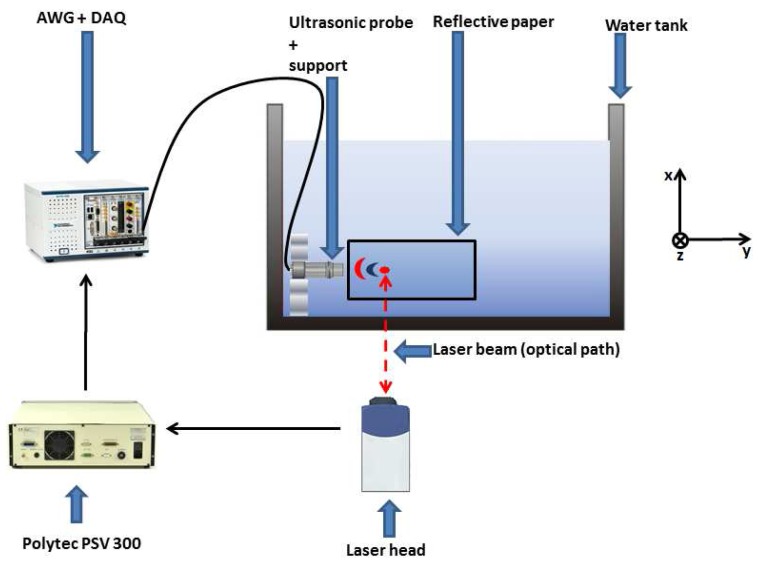
Block diagram of the experimental setup for underwater acoustic wavefront visualization. The scanning region is along the xy-plane. A matrix of 100 by 50 spatial points was acquired, corresponding to a total distance of 20 mm by 13 mm. The optical path is towards the *z* direction.

The use of LDV for acoustic wavefronts visualization offers several advantages. First of all, it provides the possibility to scan a region of interest with a high spatial resolution (a spatial resolution less than 1 mm with a point scan rate in the order of a few milliseconds). Moreover, it is a non-invasive approach, which means that it does not perturb the acoustic field during the scanning. The physical principle governing the measurement system is the Doppler effect occurring when laser light is scattered by a moving target. The measured velocity *v* not only depends on the optical path *z* of the laser beam (see Figure 1 for the axes orientation), but also on the refraction index *r* of the medium through which the beam passes [21], according to the formula:

(1) $$v\left(y,x,t\right)=\frac{d[r(y,x,t)z]}{dt}=z\frac{dr(y,x,t)}{dt}+r\left(y,x,t\right)\frac{dz}{dt}$$

A typical setup for LDV measurements of acoustic fields includes a rigid reflector placed at a certain distance from the aquarium glass plate, behind the sound source. As the purpose is to measure the variations of the refraction index *r*, the reflecting target is kept steady, and the second term in Equation (1) disappears. In a single measurement point, the laser beam crosses the glass plate at a certain point, and it is reflected at another one situated in the rigid reflector. By scanning across an area, a 2D measurement of the acoustic field can be obtained [14,22].

## 2. Method

The experimental procedure to obtain wavefront visualizations of a focused transducer is described here, with the aim to estimate its main properties, such as focal length and beam diameter. An overview of these properties is given in Appendix A. In this section, the post-processing technique to filter undesired reflections will be briefly presented.

### 2.1. Experimental Setup

The experimental setup is composed of a 5-slot PXI chassis NI PXI-1033 with an integrated MXI-Express controller, involving an arbitrary waveform generator (AWG) NI PXI-5412 (14-bit resolution), a data acquisition system (DAQ) NI PXI-5105 (digital scope, 8 channels; 12-bit resolution), two DC generators NI PXI-4110, the LDV and the focused transducer to test. The LDV is the Polytec Scanning System PSV 300, and the output signal is acquired by the DAQ connected to the PSV 300 decoder (displacement output). The focused transducer is a Panametrics V303 with a center frequency of 1 MHz and a diameter of 13 mm. The DC generators were connected to the laser head mirrors, in order to allow the movement of the laser beam. Notice that the AWG and the DAQ used the same sampling clock (the controller clock, sampling frequency 20 MHz) with the aim of reducing the leakage errors. Moreover, the integration of the AWG and the DC generators in the same chassis resulted in an additional gain in terms of time and system stability, since the use of different ports at the same time could lead to communication conflicts. The AWG was programmed to send out a chirp signal (2500 time points, initial frequency: 10 kHz, final frequency: 2 MHz, signal period: 100 μs) to the focused immersion transducer. A reflective tape was glued directly on the back surface of the water tank, as the vibrations of the tank walls induced by the presence of the acoustic field were found negligible at the frequency range discussed in this paper. The area measured by the laser was situated in front of the transducer in the xy-plane, as illustrated in Figure 1. The point-to-point scan consisted of a total of 5000 points: 100 points in the y-direction (corresponding to approximately 20 mm) and 50 points in the x-direction (corresponding to 13 mm). Eventually, care was taken to place the ultrasonic probe far enough from the laser head, in order to reduce the effect of integrating different wavefronts along the optical path.

### 2.2. The Post-Processing Technique

**Figure 2:**
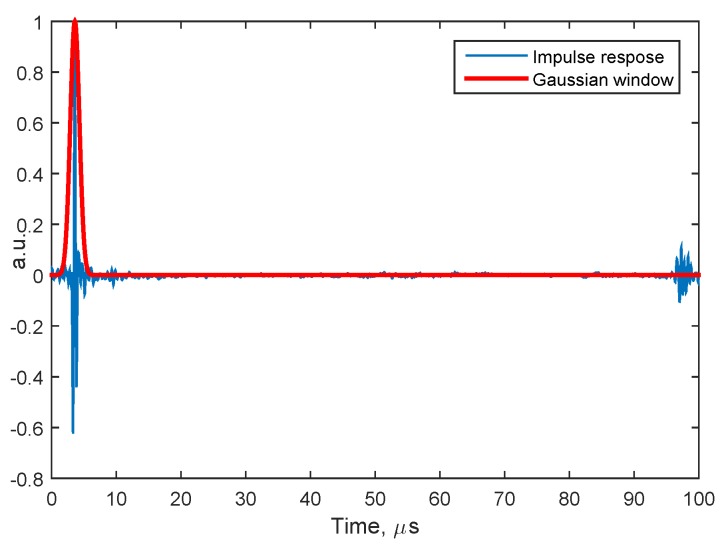
The real part of the system impulse response in a given spatial point acquired by the laser. The Gaussian window (in red) allowed filtering undesired multiple reflections.

As mentioned in the Introduction, one of the biggest issues while working in water is the presence of undesired multiple reflections within the water tank in the acquired signals, mainly due to the low attenuation coefficient of the medium. During the measurement session, we were able to acquire the signal sent to the transducer (x(t) in Equation (2)) and the one measured by the laser (y(t) in Equation (2)), for every spatial point (see Section 3 for more details). This procedure gave us the possibility to calculate the impulse response h(t) of our system, following Equation (2):

(2) $$h\left(t\right)={ℒ}^{-1}\left\{ℒ\left[y\left(t\right)\right]/ℒ\left[x\left(t\right)\right]\right\}$$

where *ℒ* indicates the Fourier transform operator. The Fourier transform was implemented in practice using the fast Fourier transform (FFT) algorithm, available in MATLAB (the MathWorks Inc., Natick, MA, USA). As example, Figure 2 shows the real part of the impulse response function in a given spatial point acquired by the laser. The use of the Gaussian window allowed an easy filtering of undesiredmultiple reflections.

**Figure 3:**
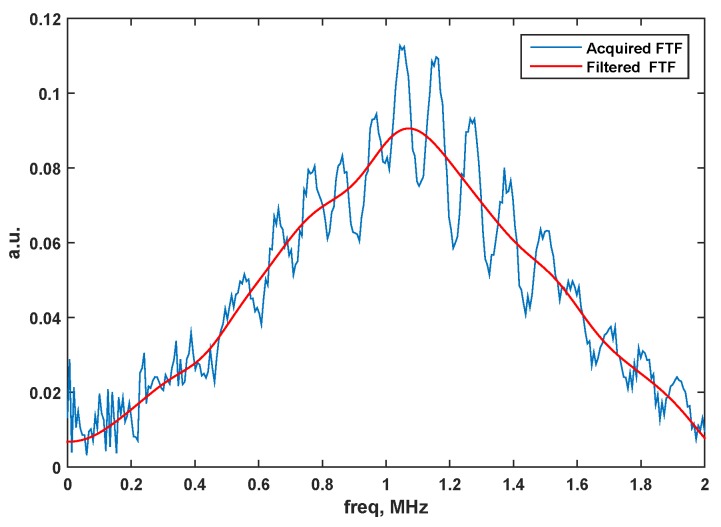
The acquired frequency transfer function (FTF) (blue) and the filtered one (red) at a given spatial point.

The filtered data have been used to calculate the frequency transfer function (FTF) for all of the acquired spatial points. In example, Figure 3 depicts the acquired FTF and the filtered one at a given spatial point. One can note that oscillations appeared in the initial FTF, as multiple reflections were present in the corresponding time domain signal. These oscillations were not present anymore in the filtered one.

## 3. Results and Discussion

Before showing the final results of the described procedure, important aspects concerning underwater measurements still need to be addressed. First of all, one should take into account the small wavelengths of the acoustic waves in water (approximately 0.75 mm at 2 MHz). As a consequence, the number of points to acquire will be quite large, in order to obtain an accurate spatial reconstruction. Thus, a new system able to scan the surface of interest in a relatively short time will play an important role during the experiments, as conventional systems generally can take up to three hours to acquire a few thousand spatial points. Secondly, the size of the laser beam should be smaller than the smallest wavelength composing the excitation signal, in order to measure the real value of the wave and not an averaged one.

To counter the first problem, at every point, we acquired simultaneously the signal sent to the transducer and the one measured by the LDV. This not only gave us the possibility to filter the data via the impulse response function (see Section 2.2), but also to avoid any kind of triggering between the generator (NI-5412) and the acquisition device (NI-5105). Hence, the system became faster (approximately by a factor of two), without losing the phase information of the acquired waves. The second problem was solved adding a close-up unit to the laser scanning head in order to obtain a very focused beam.

Another time-consuming operation is the averaging part, which is generally required if one wants to increase the SNR of the acquired signals. This time loss is mainly due to the communication between the instrumentation and the PC. To reduce it, we acquired a large number of periods at once (the number of periods is equal to the number of averages). As a result, a measurement session composed of a time signal of 2500 samples (sampling frequency of 20 MHz), acquired in 5000 spatial points, required only about thirty minutes.

In the next paragraphs, the ultimate results of this work will be presented. As stated earlier, the main objective is to visualize the wavefronts generated by the focused transducer under test.

### 3.1. Acoustic Wavefront Visualizations

**Figure 4:**
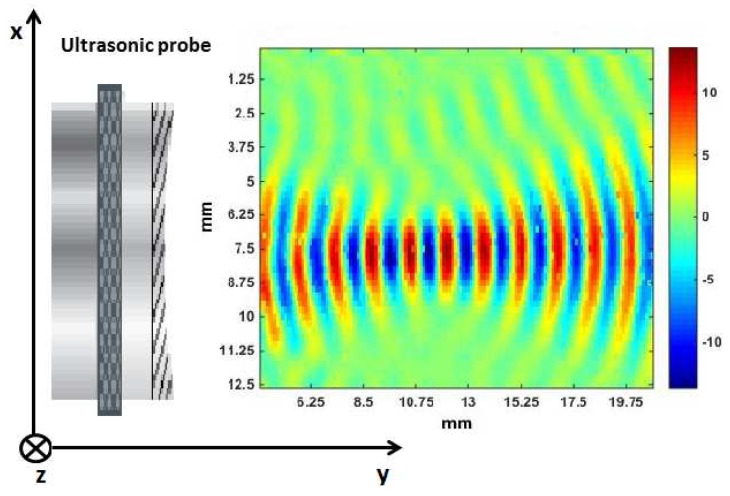
Laser Doppler vibrometry (LDV) visualization of the focused transducer Panametrics V303 for the frequency of 1 MHz. The ultrasonic probe is located on the left of the first measured wavefront.

The wavefronts generated by the focused transducer Panametrics V303 using LDV measurements are depicted in Figure 4. The colors in Figure 4 represent the scaled real part of the frequency transfer function obtained in Section 2.2, at a frequency of 1 MHz and reshaped in all of the spatial points measured by the LDV. This allowed us to clearly identify the trend of the acoustic wavefronts as a function of the distance. Figure 4 also shows the transducer position, on the left of the first measured wavefront. As stated before (see also Figure 1), the area investigated by the laser was situated in the xy-plane, while the optical path was along the *z*-axis. It is interesting to note that, within the focal region, the diameter of the sound beam remains almost constant. As a consequence, considering only this region could allow one the use of a plane wave model to describe the wave propagation.

**Figure 5:**
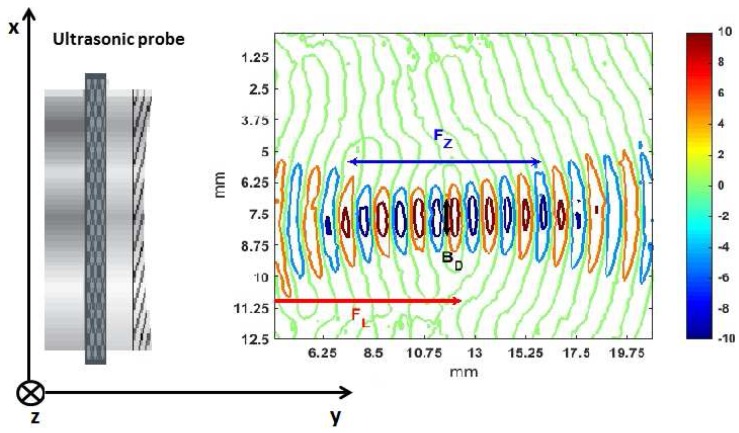
Topographical view of the wave propagation shown in Figure 4. The arrows indicate the focal length $F_{L}$, the focal zone $F_{Z}$ and the beam diameter $B_{D}$ at the focus point.

In order to better show the evolution of the beam diameter, we propose in Figure 5 a topographical view of the wave propagation shown in Figure 4.

From Figure 4 and Figure 5, it is possible to obtain the main parameters describing the sound beam, already mentioned in the Introduction. The focal length $F_{L}$, the focal zone $F_{Z}$ and the beam diameter at the focus point $B_{D}$ were calculated taking into account different zones of the wavefronts (the central points, points at the borders). The mean value of these distances appears in the third column of Table 1 followed by standard deviations given in parentheses. These results can be compared with the data sheet specification of the transducer and the theoretical values obtained with Equations (A1)–(A3) using *D* = 13 mm, *f* = 1 MHz and $v_{M}$ = 1500 m/s.

**Table 1:** Experimental and theoretical sound beam parameters for Panametrics V303. The experimental values have been determined from Figure 5. The standard deviations are given in parentheses.

Parameter	Theoretical Value, mm	Experimental Value, mm
$F_{L}$	12.8 *	12 (1.4)
$F_{Z}$	9.5 **	9.5 (0.7)
$B_{D}$	1.6 ***	1.7 (0.3)

* From the transducer data sheet; ** from Equations (A1) and (A2); *** from Equation (A3).

As shown in Table 1, the estimated sound beam parameters are almost the same as the reference values given by the probe data sheet and the analytical values obtained through Equations (A1)–(A3). Even if it were possible to estimate at least $F_{L}$ by only aiming the sound beam at a reflector and moving the probe until the largest echo is measured, our method based on LDV measurements gave in addition a global view of the wave propagation and the possibility to estimate at once all of the sound beam parameters. Moreover, thanks to the wavefront visualization, we had the opportunity to observe another interesting property of the focused sound beam, as explained in the next paragraph.

### 3.2. Attenuation and Inhomogeneities in the Ultrasound Field

**Figure 6:**
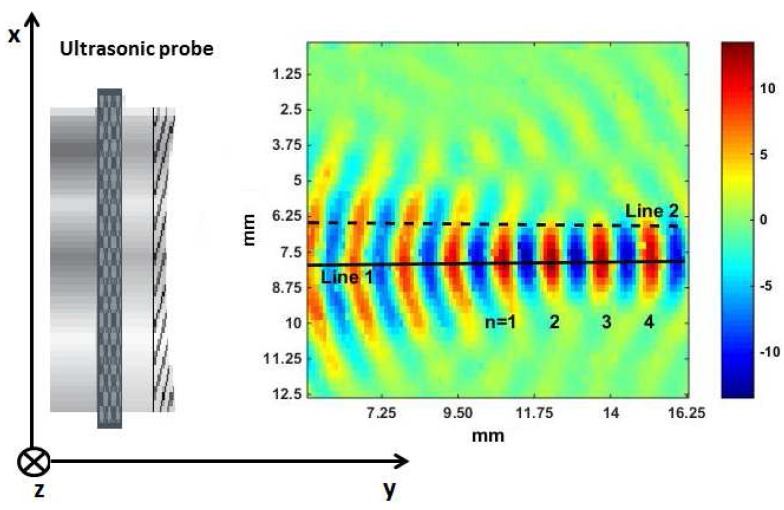
LDV visualization of the focused transducer Panametrics V303 for the frequency of 1 MHz. Line 1 indicates points situated in the center of the beam diameter, while Line 2 represents points at the beam border. The variable *n* indicates wavefronts situated in the focal region.

In this section, we investigate in more detail the properties of the sound beam generated by the focused transducer Panametrics V303, based on LDV measurements. To do so, a laser scan over a restricted area (corresponding to approximately 16 mm in the y-direction and 13 mm in the x-direction) has been performed. The results are depicted in Figure 6. Afterwards, two lines (Line 1 and Line 2) were drawn in Figure 6, as interesting information about the variations of the signals frequency content as a function of the spatial position can be deduced at certain key points on both lines.

**Figure 7:**
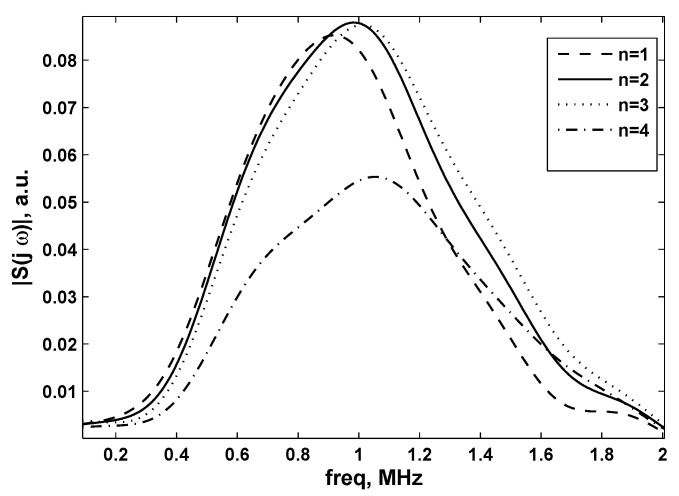
Fourier transform absolute values of signals situated at the center of the sound beam (Line 1) for different values of *n* (see also Figure 6).

**Figure 8:**
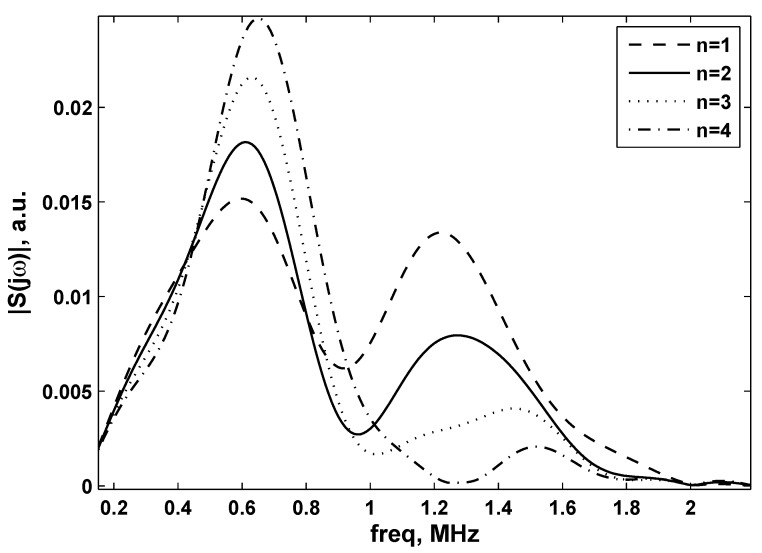
Fourier transform absolute values of signals situated at the sound beam border (Line 2) for different values of *n* (see also Figure 6).

These variations can be highlighted calculating the absolute value of the FFT of different signals recorded along Line 1 and Line 2, indicated in Figure 7 and Figure 8 as |S(jω)|, with *j* the imaginary unit and *ω* the angular frequency.

From Figure 7, it can be concluded that the peak at the central frequency (1 MHz) appears at *n* = 2, which is in accordance with the following facts: high frequencies are more directive and the transducer reaches the highest amplitude (*i.e.*, on the central frequency) only at the focal point. The points belonging to spatial positions outside the focal region (*i.e.*, *n* = 4 in Figure 6), registered an attenuated peak at the central frequency of the transducer.

On the other hand, in Figure 8, the Fourier transform of signals at certain key locations situated in Line 2 (the border of the sound beam) is depicted. From this figure, it is clear that frequencies around the central one (1 MHz in this case) almost disappear, for every value of *n*. This could lead to the conclusion that the focused transducer is able to distribute energy over the spectra lines around the central frequency only at the center of the sound beam. Taking into account the studies reportedin [23,24,25,26,27,28], these inhomogeneities at the border of the ultrasound field could be due to the finite aperture of the probe and interpreted as diffraction effects.

## 4. Conclusions

In this paper, we performed LDV measurements to visualize the acoustic field generated by a focused ultrasonic transducer, immersed in a water tank. A high-speed experimental setup, together with a dedicated filtering technique, has been developed in order to obtain reliable information about the generated sound beam in a short length of time (approximately thirty minutes). The use of LDV offered several advantages, such as the possibility to scan the region of interest in a non-invasive way and with high sensitivity. Inhomogeneities at the borders of the sound beam have been observed. Even if a link between these phenomena and diffraction effects has been proposed, more investigations are planned for the future. Eventually, the work described in this paper could easily be extended to other typesof transducers.

## Appendix

### A. Most Common Focused Sound Beam Parameters

In this paragraph, the properties of a focused sound beam are briefly mentioned. As reported in [29], the length of a transducer focal zone is given by Equation (A1):

(A1) $$F_{Z}=NS_{F}^{2}\frac{2}{1+0.5S_{F}}$$

with $F_{Z}$ = Focal zone, $S_{F}=F_{L}/N$ (normalized focal length). The near field *N* is calculated as:

(A2) $$N=\frac{D^{2}f}{0.5v_{M}}$$

with *f* = frequency, *D* = element diameter and $v_{M}$ = material sound velocity. Another important parameter for the sound beam is the beam diameter, which mainly affects the transducer's sensitivity at the point of interest. The −6 dB pulse-echo beam diameter ($B_{D}$) at the focus can be calculated with Equation (A3) or Equation (A4). For a flat transducer, use Equation (A4) with $S_{F}$ = 1.

(5) $$B_{D}\left(-6\;dB\right)=1.02\frac{F_{L}v_{M}}{fD}$$

(6) $$B_{D}\left(-6\;dB\right)=0.2568DS_{F}$$
